# *Dicranopteris linearis* extract inhibits the proliferation of human breast cancer cell line (MDA-MB-231) via induction of S-phase arrest and apoptosis

**DOI:** 10.1080/13880209.2018.1495748

**Published:** 2018-10-10

**Authors:** Aifaa Akmal Baharuddin, Rushduddin Al Jufri Roosli, Zainul Amiruddin Zakaria, Siti Farah Md. Tohid

**Affiliations:** a Halal Products Development, Halal Products Research Institute, Universiti Putra Malaysia (UPM)Serdang, Selangor, Malaysia;; b Department of Biomedical Science, Faculty of Medicine and Health Sciences, Universiti Putra Malaysia (UPM)Serdang, Selangor, Malaysia

**Keywords:** Anticancer, antiproliferative, acridine orange propidium iodide, annexin-V FITC, cell cycle arrest

## Abstract

**Context:***Dicranopteris linearis* (Burm.f.) Underw. (Gleicheniaceae) has been scientifically proven to exert various pharmacological activities. Nevertheless, its anti-proliferative potential has not been extensively investigated.

**Objective:** To investigate the anti-proliferative potential of *D. linearis* leaves and determine possible mechanistic pathways.

**Materials and methods:** MTT assay was used to determine the cytotoxic effects of *D. linearis* methanol (MEDL) and petroleum ether (PEEDL) extracts at concentrations of 100, 50, 25, 12.5, 6.25 and 3.125 µg/mL against a panel of cancer cell lines (breast [MCF-7 and MDA-MB-231], cervical [HeLa], colon [HT-29], hepatocellular [HepG2] and lung [A549]), as compared to negative (untreated) and positive [5-fluorouracil (5-FU)-treated] control groups. Mouse fibroblast cells (3T3) were used as normal cells. The mode of cell death was examined using morphological analysis via acridine orange (AO) and propidium iodide (PI) double staining. Cell cycle arrest was determined using flow cytometer, followed by annexin V-PI apoptosis detection kit.

**Results:** MEDL demonstrated the most significant growth inhibition against MDA-MB-231 cells (IC_50_ 22.4 µg/mL). PEEDL showed no cytotoxic effect. Induction of apoptosis by MEDL was evidenced via morphological analysis and acridine orange propidium iodide staining. MEDL could induce S phase cell cycle arrest after 72 h of incubation. Early apoptosis induction in MDA-MB-231 cells was confirmed by annexin V-FITC and PI staining. Significant increase in apoptotic cells were detected after 24 h of treatment with 15.07% cells underwent apoptosis, and the amount escalated to 18.24% with prolonged 48 h incubation.

**Conclusions:** MEDL has potential as a potent cytotoxic agent against MDA-MB-231 adenocarcinoma.

## Introduction

Breast cancer is one of the most frequently diagnosed cancers and the leading cause of cancer death in women with an estimate of 1.7 million cases and 521,900 deaths worldwide in 2012 (Torre et al. 2015). It is undeniable that the current chemotherapy is effective in treating cancer patients; however, the presence of undesirable adverse effects has triggered the demand for new therapeutic agents. Therefore, continuous efforts are being made in the quest for a novel bioactive compound to combat breast carcinomas. At present, bioactive compounds of natural sources, particularly from plants which have been traditionally used, are gaining significant attention. *Dicranopteris linearis* (Burm.f.) Underw (Gleicheniaceae), locally known to the Malays as ‘resam’ is a type of fern found in secondary forest. The leaves of *D. linearis* have been used in Malay traditional medicine to reduce body temperature and control fever (Chin 1992; Derus 1998). Several investigations have demonstrated that the plant extracts of *D. linearis* possess numerous health-promoting properties such as antinociceptive, anti-inflammatory, anti-pyretic (Zakaria et al. 2008), potential cytotoxic and antioxidant activities against various types of cancer (Zakaria et al. 2011). In this study, two different types of extracts from *D. linearis* were analyzed to investigate their cytotoxicity activities against several cancer cell lines. The type of extract that showed the best cytotoxic activity on the most susceptible cancer cell line was then chosen for further examination in order to delineate the mode of death and cell cycle arrest.

## Materials and methods

### Chemicals

3-(4,5-Dimethylthiazol-2-yl)-2,5-diphenyltetrazolium bromide (MTT), 5-fluorouracil (5-FU), Dulbecco’s modified Eagle’s medium (DMEM), fetal bovine serum (FBS), Roswell Park Media Institute (RPMI) 1640 and penicillin–streptomycin solution were purchased from Sigma–Aldrich (St. Louis, MO, USA). The Annexin V-FITC Apoptosis Detection Kit and CycleTEST^TM^ PLUS DNA Reagent Kit were purchased from BD Pharmingen (San Diego, CA, USA). All other chemicals used were of analytical grade.

### Plant material

The leaves of *D. linearis* were collected between November to December 2014 from their natural habitat at the Universiti Putra Malaysia, Serdang, Selangor, Malaysia. Authentication was done by Dr. Shamsul Khamis at the Biodiversity Unit, Institute of Bioscience, Universiti Putra Malaysia. A voucher specimen was deposited as SK 2680/15. The leaves of *D. linearis* were washed, rinsed and oven-dried at a temperature of 37 °C. The leaves were then removed from the stem and ground into a coarse powder form using an electric grinder (RT Precision Technology Co., Taichung City, Taiwan). The coarsely powdered leaves were stored at room temperature.

### Preparation of methanol (MEDL) and petroleum ether (PEEDL) extracts of *D. linearis*

The extracts of *D. linearis* methanol (MEDL) and petroleum ether (PEEDL) were prepared following the protocol previously described by Zakaria et al. (2011). The coarsely powdered leaves (10 g) were soaked in 200 mL of methanol (MeOH) or petroleum ether (PE) at the ratio of 1:20 (w/v) for 72 h at room temperature. Insoluble materials were filtered using a steel filter and cotton wool, followed by a filter paper (Whatman No.1). The filtrate was concentrated through evaporation under reduced pressure using a rotary evaporator machine (Heidolph Instruments GmbH & Co., Schwabach, Germany) at 40 °C until dried and yielded 3.52 g of MEDL and 0.15 g of PEEDL. The initial stock solutions were prepared by dissolving 100 mg of MEDL in 1 mL of dimethyl sulfoxide (DMSO) and 100 mg of PEEDL in 1 mL of absolute ethanol to give 100 mg/mL of stock solutions. Next, both the MEDL and PEEDL solutions were further diluted using serial dilution to get final treatment concentrations ranging between 100 to 3.12 µg/mL. The final concentrations of MEDL contained less than 0.1% of DMSO, and PEEDL contained less than 0.1% of ethanol. Under these conditions, DMSO and ethanol were not toxic to any cell lines used in this study.

### Preparation of 5-fluorouracil (5-FU)

The stock solution was prepared by dissolving 10 mg of 5-FU in 1 mL of fresh media. Then, 5-FU was further diluted using a serial dilution to get the final 5-FU concentrations ranging between 100 and 3.12 µg/mL (Li et al. 2004).

### Preparations of cell lines and cell culture

The cell lines used in this study were breast adenocarcinomas (MCF-7 and MDA-MB-231), cervical adenocarcinoma (HeLa), colon carcinoma (HT-29), hepatocellular carcinoma (HepG2), lung carcinoma (A549) and normal mouse fibroblast cells (3T3). A549, HeLa, HepG2, MCF-7 and 3T3 cells were cultured in RPMI 1640 containing 10% FBS, and 1% penicillin–streptomycin. The HT29 and MDA-MB-231 cells were cultured in DMEM containing 10% FBS and 1% penicillin–streptomycin. All cells were cultured in an incubator at 37 °C with 95% humidity and 5% CO_2_.

### MTT cytotoxic assay

The cytotoxic activity of the extracts (MEDL and PEEDL) and 5-FU was examined by using a MTT assay. The procedures were performed as reported by Zakaria et al. (2011) with a slight modification, as explained here. The cytotoxic activity of MEDL and PEEDL was tested against A549, HeLa, HepG2, HT29, MCF-7 and MDA-MB-231 while the cytotoxic activity of 5-FU was tested only against MDA-MB-231 cells. Cell suspension (100 µL) with a density of 1.0 × 10^5^ was seeded in a 96-well flat-bottomed plate. After 24 h incubation at 37 °C in 95% humidity and 5% CO_2_, the culture medium was removed from the wells, and 100 µL of the extracts/drug (at 100, 50, 25, 12.5, 6.25, and 3.125 µg/mL) was added to each well and incubated for another 72 h (initial method used 100 to 12.5 µg/mL extracts/drug). After 72 h of post-treatment, the medium in each well was aspirated and replaced with 100 µL of media, and 20 µL of MTT solution (MTT salt mixed with PBS to attain the final concentration of 5 mg/mL) was added to each well and incubated for an additional 3 h in 5% CO_2_ humidified incubator at 37 °C. Formazan crystals were dissolved by adding 100 µL of DMSO. The amount of purple formazan produced, which was relative to the number of viable cells, was determined by measuring the absorbance with ELISA plate reader (Biochrom Asys UVM 340, San Francisco, CA, USA) at a wavelength of 570 nm (initial method used 550 nm wavelength). All assays were carried out in triplicate and in three independent tests. The percentage of cytotoxic activity compared to that of untreated cell was determined as follows:

$$\text{Cell}\;\text{viability}\left(\%\right)=\frac{\left(\text{OD}\;\text{sample}–\text{OD}\;\text{blank}\right)}{\left(\text{OD}\;\text{control}–\text{OD}\;\text{blank}\right)}\times 100\%$$

### Optimization of time period in MTT cytotoxicity assay

In order to determine the optimization period of the cytotoxic activity of MEDL against MDA-MB-231 cells, a slightly modified MTT cytotoxicity assay following a protocol from Alabsi et al. (2012) was carried out. Cells were seeded at a density of 1 × 10^5^ (initial method used 5 × 10^5^ cell density) in 100 µL media in a 96-well plate, and allowed to incubate for 24 h for cell attachment. Then, the cells were exposed to the IC_50_ concentration of MEDL (22.4 µg/mL) and incubated at 37 °C under 5% CO_2_ for 24, 48 and 72 h (initial method used both IC_50_ and IC_25_ concentrations). Each sample was assayed in triplicate. At the end of the incubation periods, the medium was aspirated off and replaced with 100 µL of media and 20 µL of 5 mg/mL MTT solution and incubated at 37 °C in an atmosphere of 5% CO_2_ for another 3 h. Formazan crystals were dissolved by adding 100 µL of DMSO. The amount of purple formazan produced relative to the number of viable cells was determined by measuring the absorbance with ELISA plate reader (Biochrom Asys UVM 340, San Francisco, CA, USA) at a wavelength of 570 nm. The percentage of anti-proliferative activity compared to that of untreated cells was determined as follows:

$$\text{Cell}\;\text{viability}\left(\%\right)=\frac{\left(\text{OD}\;\text{sample}–\text{OD}\;\text{blank}\right)}{\left(\text{OD}\;\text{control}–\text{OD}\;\text{blank}\right)}\times 100\%$$

### Morphological analysis of MDA-MB-231 cells

MDA-MB-231 cells were seeded at 1 × 10^5^ cells (initial method used 2 × 10^4^ cells) in 4 mL medium in a 25 cm^3^ flask (initial method used petri plate to seed the cells). After 24 h of incubation, the medium in each well was removed and replaced with MEDL at IC_50_ concentration (22.4 µg/mL) for 24, 48, and 72 h (initial method used 24 h incubation only). At the end of the treatment, the effect of MEDL on morphological changes of MDA-MB-231 cells was assessed using a phase-contrast microscope (CK40, Olympus Optical Co., Ltd., Tokyo, Japan) at 200× and 400× magnifications (initial method used 100× magnification only). Procedures were obtained from Srivastava et al. (2011) with slight modification, as detailed here.

### Acridine orange (AO) and propidium iodide (PI) double staining using fluorescent microscopy

MDA-MB-231 cells were quantified using acridine orange (AO) and propidium iodide (PI) double staining according to standard procedures and examined under fluorescence microscope (Leica DM 2500, Leica Microsystems, Wetzlar, Germany). Cells were seeded at 1 × 10^5^ cells (initial method used 5 × 10^5^ cells) in 4 mL medium in a 25 cm^3^ flask (initial method used 6-well plate to seed the cells). After 24 h of incubation, the medium in each well was removed and replaced with IC_50_ concentrations of MEDL (22.4 µg/mL) and 5-FU (19.88 µg/mL). After 24, 48, and 72 h of incubation, the treated and untreated cells were washed with 1 mL PBS, trypsinized and centrifuged at 1000 rpm for 5 min. The supernatant was discarded to obtain a cell pellet. The cell pellet was mixed with an equal volume of staining solution, at a ratio of 1:1 containing 1 mg/mL AO and 1 mg/mL PI (dissolved in PBS) and incubated in the dark for 10 min (initial method used 10 µg/mL AO and PI, respectively). Stained cell suspension (10 µL) was dropped onto a glass slide and covered with a cover slip. Cells were observed under a fluorescence microscope at 400× magnification within 30 min before the fluorescence faded. Assays were carried out in triplicate and in three independent tests. Viable, apoptotic and necrotic cells were quantified in a population of 200 cells. The results were expressed as a proportion of the total number of the cells examined. Procedures were obtained from Alabsi et al. (2012) with slight modification, as detailed here.

### Determination of cell cycle arrest by flow cytometer

A cell cycle assay was performed using the BD Pharmingen CycleTEST^TM^ PLUS DNA Reagent Kit (San Diego, CA, USA) following the manufacturer’s protocol. Cells at a density of 5 × 10^5^ in 4 mL medium were seeded in a 25 cm^3^ flask. Cells were then treated with IC_50_ values of MEDL and 5-FU for 24, 48, and 72 h. After the incubation period, all adhering and floating cells were harvested and transferred into sterile centrifuge tube. Cells were centrifuged at 400*g* for 5 min at room temperature. The supernatant was aspirated, leaving approximately 50 µL of residual fluid in the tube to avoid disturbing the pellet. Trypsin buffer (250 µL) was added into each centrifuge tube containing the cell pellet, and gently mixed by tapping the tube with fingers. The mixtures were then incubated for 10 min at room temperature. Then, 200 µL of trypsin inhibitor and RNase buffer were added into each tube, gently mixed and incubated for 10 min at room temperature. Cold PI stain solution (200 µL) was added into each tube and was gently mixed. The mixtures were incubated for 10 min on ice (2° to 8 °C) in the dark. The samples were then filtered through a 50 µm nylon mesh into a 12 × 75 mm tube. The samples were tested by using the BD FACSCanto II flow cytometer (BD Biosciences, San Jose, CA, USA) within an hour and analyzed using ModFit LT 3.2 Software (BD Biosciences, San Jose, CA, USA).

### Determination of mode of cell death using Annexin V-propidium iodide

The mode of cell death induced by MEDL was evaluated using the BD Pharmingen Annexin V-FITC Apoptosis Detection Kit (San Diego, CA, USA) following the manufacturer’s protocol. Cells at a density of 5 × 10^5^ cells in 4 mL medium were seeded in a 25 cm^3^ flask. The cells were then treated with IC_50_ values of MEDL and 5-FU for 24 and 48 h. After the incubation period, all adhering and floating cells were harvested and transferred into sterile centrifuge tubes. The cells were centrifuged at 400*g* for 5 min at room temperature. The supernatant was aspirated, leaving approximately 50 µL of residual fluid in the tubes to avoid disturbing the pellets. FITC Annexin V (5 µL) and PI (5 µL) were added into the tubes containing cell pellets. The mixtures were gently vortexed and incubated for 15 min at room temperature in the dark. Binding buffer (400 µL) was added into each tube. The samples were analyzed using the BD FACSCanto II flow cytometer (BD Biosciences, San Jose, CA, USA) within an hour.

### Statistical analysis

Data are presented as mean ± standard error of the mean (SEM) of three independent experiments. Statistical analysis was performed using GraphPad Prism version 6.0 software (GraphPad Software, Inc., La Jolla, CA, USA). One way analysis of variance (ANOVA) and *post hoc* Tukey’s multiple comparison tests were used to determine the differences among the means. Value of *p <* 0.05 was considered to be statistically significant.

## Results and discussion

### Cytotoxic effects of *D. linearis* extracts on cancer cell lines and normal mouse fibroblast cells

The results of the study revealed growth inhibition activities of *D. linearis* extracts, namely, MEDL and PEEDL on several types of cancer cells including breast adenocarcinomas (MCF-7 and MDA-MB-231), cervical adenocarcinoma (HeLa), colon carcinoma (HT-29), hepatocellular carcinoma (HepG2) and lung carcinoma (A549), as well as on normal mouse fibroblast cells (3T3). MEDL and PEEDL were prepared in different concentrations (100, 50, 25, 12.5, 6.25, 3.125 µg/mL), and the cell viability was analyzed using MTT assay in three independent experiments. Previous study performed by Zakaria et al. (2011) revealed that aqueous extract of *D. linearis* failed to show any sign of growth inhibition against numerous cancer cells that were used in the study. Therefore, the anti-proliferative screening using aqueous extract was not carried out, as this study was intended to determine only the cytotoxic effects of MEDL and PEEDL.

**Figure 1.**
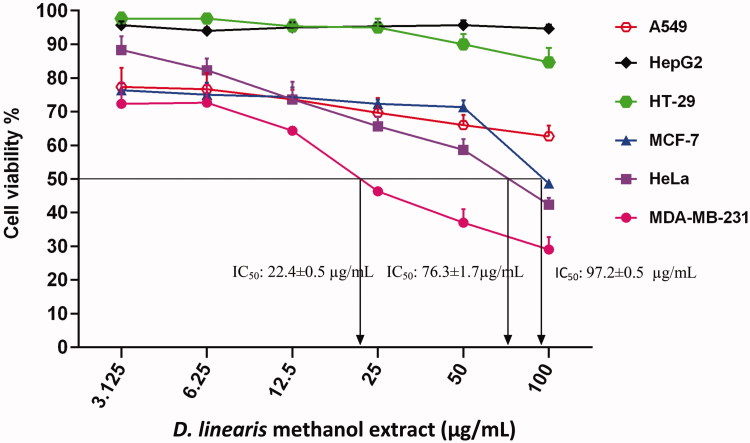
The concentration–response curve of the cancer cell lines derived from MTT cytotoxicity assay performed after 72 h exposures with MEDL. Data are presented as mean ± standard error (*n* = 3).

**Figure 2.**
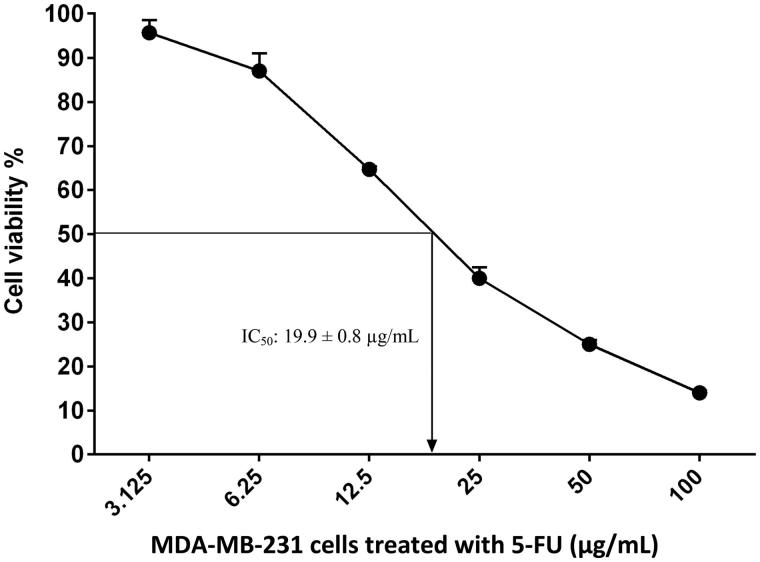
The concentration–response curve of MDA-MB-231 cancer cell line derived from MTT cytotoxicity assay performed after 72 h exposures with 5-FU. Data are presented as mean ± standard error (*n* = 3).

Results showed that MEDL was effective against MDA-MB-231, HeLa and MCF-7 cancer cells with IC_50_ values recorded at 22.4 ± 0.5, 76.3 ± 1.7, and 97.2 ± 0.5 µg/mL, respectively (Figure 1). Among these three cancer cells, MDA-MB-231 cells exhibited the most potent growth inhibitory activity when tested with MEDL, and therefore were chosen to be further tested against positive control 5-FU, with the IC_50_ value recorded at 19.9 ± 0.8 µg/mL (Figure 2).

**Figure 3.**
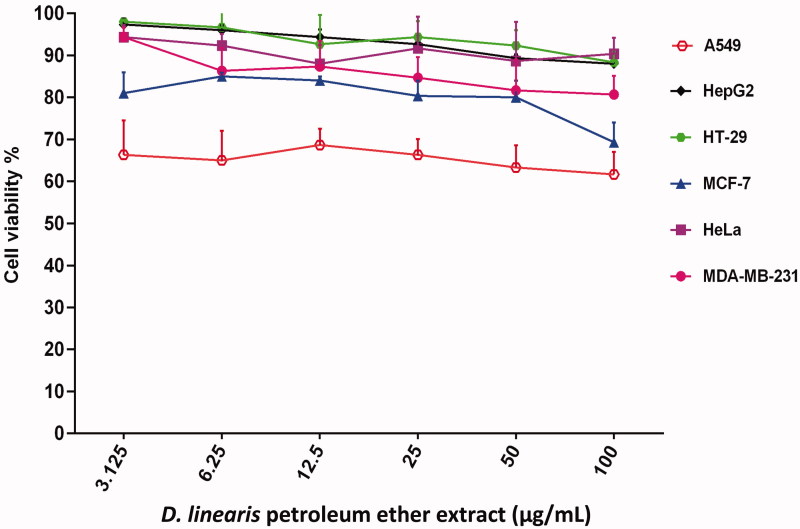
The concentration–response curve of the cancer cell lines derived from MTT cytotoxicity assay performed after 72 h exposures with PEEDL. Data are presented as mean ± standard error (*n* = 3).

**Figure 4.**
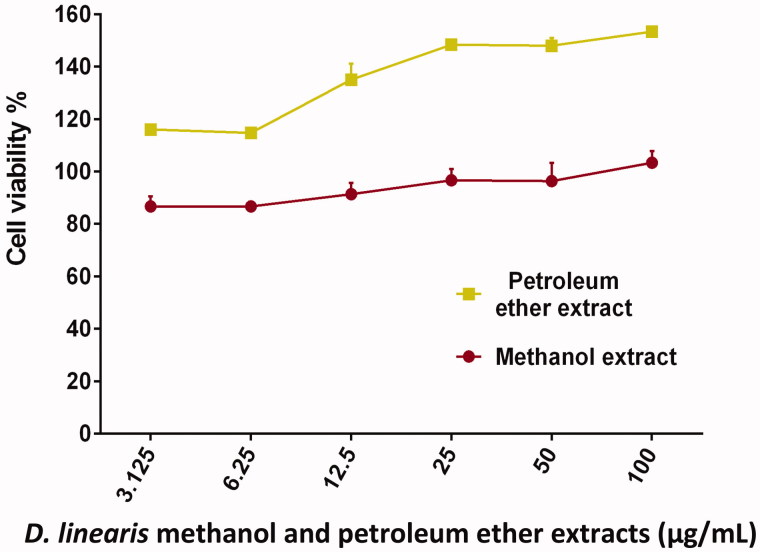
The concentration–response curve of the 3T3 cell line derived from MTT cytotoxicity assay performed after 72 h exposures with MEDL and PEEDL. Data are presented as mean ± standard error (*n* = 3).

On the other hand, PEEDL which is a non-polar extract failed to inhibit the growth of any type of cancer cells when treatment was given at 100 µg/mL (Figure 3). Subsequently, both MEDL and PEEDL did not show cytotoxic activity on normal mouse fibroblast cells (3T3) even at the highest concentration of 100 µg/mL (Figure 4). This suggests that MEDL is selective towards the proliferating cancer cells. These cyto-selective properties of MEDL have been reported earlier by Zakaria et al. (2011). Based on the results, MEDL is suggested to have chemotherapeutic potential against cervical and breast adenocarcinomas without harming the normal cells. The results also suggested that MEDL activity was not dependent on estrogen receptor as it is more cytotoxic to the MDA-MB-231 estrogen-independent human breast cancer cells as compared to the MCF-7 estrogen-dependent human breast cancer cells.

Summary of the cytotoxic activities of MEDL, PEEDL and 5-FU is presented in Table 1. Among the two *D. linearis* extracts, MEDL was found to have the most effective IC_50_ value against MDA-MB-231 cancer cells. Therefore, the subsequent investigations were mainly focused on the apoptotic properties of MEDL against MDA-MB-231 cell line, which was the most sensitive cell line to MEDL.

**Table 1.** Data represents the mean IC_50_ values and standard error of mean (SEM) for each of the cell lines tested with MEDL, PEEDL and 5-FU by MTT Assay.

Extract		IC_50_ values (µg/mL)					
	3T3	A549	HeLa	HepG2	HT-29	MCF-7	MDA-MB-231
MEDL	ND	ND	76.3 ± 1.7	ND	ND	97.2 ± 0.5	22.4 ± 0.5**^a^**
PEEDL	ND	ND	ND	ND	ND	ND	ND
5-FU	–	–	–	–	–	–	19.9 ± 0.8

### Determination of the optimization period of the cytotoxic activity of *D. linearis* methanol extract against MDA-MB-231 cells

**Figure 5.**
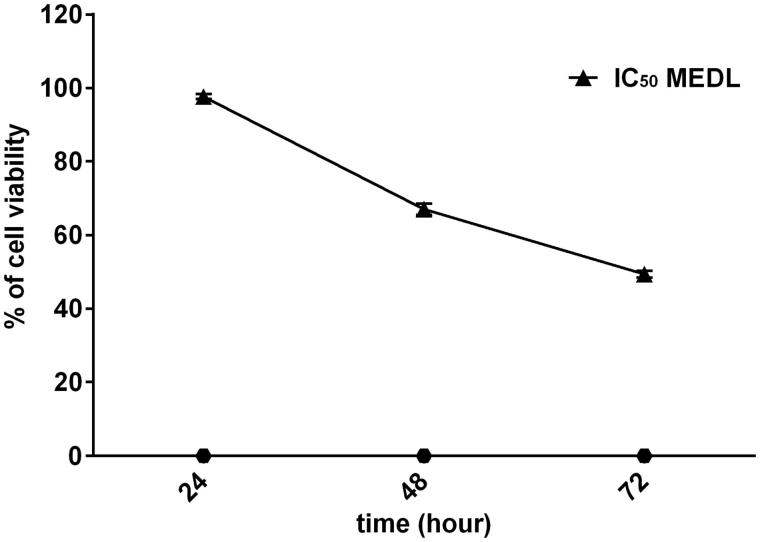
MTT cytotoxicity assay for IC_50_ (22.4 µg/mL) of MEDL against MDA-MB-231 cells at 24, 48, and 72 h post-treatment. Data are presented as mean ± standard error (*n* = 3).

MTT assay was carried out to determine the optimization period of the MEDL cytotoxic activity against MDA-MB-231 cancer cells. The MDA-MB-231 cells were treated with IC_50_ concentration of MEDL (22.4 µg/mL) and incubated for different time periods at 24, 48, and 72 h in three independent assays to detect the cell viability. The purpose was to observe whether MEDL could inhibit the growth of MDA-MB-231 cells in a time-dependent manner. Figure 5 showed the percentage of cell viability that decreased with prolonged MEDL treatment. The percentage of cell viability when incubated at 24, 48, and 72 h were recorded at 98, 67, and 49%, respectively. This shows that the growth inhibition of MEDL increases as the incubation period increases. The result also revealed that there was a time-dependent decrease in the viability of the cancer cells, and the optimal period of the cytotoxic activity of MEDL was found to be at 72 h. It is important to monitor the growth of the cancer cells in different time periods in order to avoid recurrent of the cancer cells growth during the incubation periods. Therefore, this assay has successfully revealed that MEDL has the competency to inhibit the growth of MDA-MB-231 cells in a time-dependent manner, and it is proposed that the recurrent of MDA-MB-231 cells growth does not occur throughout the incubation period of 24, 48 and 72 h.

### The effects of *D. linearis* methanol extract on the morphology of breast cancer cell line

**Figure 6.**
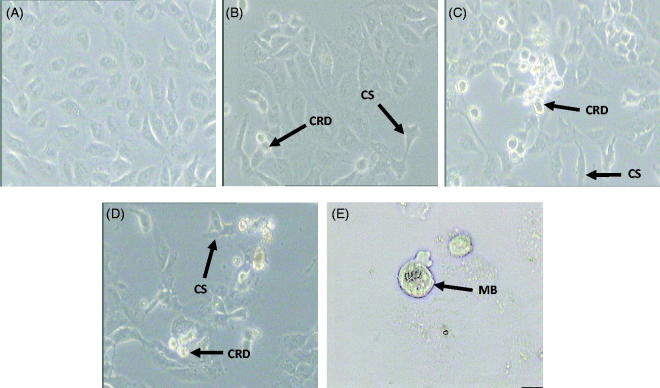
Morphological changes of MDA-MB-231 cells were examined under a phase contrast microscope. Untreated MDA-MB-231 cells (A), MDA-MB-231 cells treated with IC_50_ concentration of MEDL after 24 h (B), 48 h (C), and 72 h (D) at 200× magnification. MDA-MB-231 cells treated with IC_50_ concentration of MEDL after 72 h (E) at 400× magnification. (CRD): cell rounding and detachment; (CS): cell shrinkage; (MB): membrane blebbing. Experiment was completed in three independent experiments in triplicate.

The results of MTT optimization assay of MEDL against MDA-MB-231 cells were further supported by morphological study using a phase contrast microscope in three independent experiments to identify apoptotic cells. In this assay, only morphological changes of the MDA-MB-231 cells treated with the IC_50_ concentration of MEDL were studied as a way to confirm that MEDL could induce apoptotic cell death. Cells were divided into untreated group and groups treated with IC_50_ concentration of MEDL for 24, 48, and 72 h incubation periods. Apoptotic cells are characterized by cell shrinkage, membrane blebbing, chromatin condensation, nuclear and DNA fragmentation, and breaking up of cells into a membrane-bounded vesicle, known as ‘apoptotic bodies’, which are consequently ingested by macrophages (Doyle and Griffiths 1998; Nagasaka et al. 2010). It is important to determine the mode of cell death induced by MEDL since cell death can be divided into apoptosis or necrosis. Apoptosis is the desired mode of cell death because it is a well-organized, programmed cell death that does not trigger inflammatory responses (Millan and Huerta 2009). Results showed that changes such as cell shrinkage, cell rounding, cell detachment and membrane blebbing were observed in MDA-MB-231 cells as they underwent apoptosis after being treated with MEDL (Figure 6). These morphological changes were consistent with the apoptotic morphology characterized by Doyle and Griffiths (1998) and Nagasaka et al. (2010), thus suggested that MEDL may induce apoptotic cell death in MDA-MB-231 cells.

### Qualitative and quantitative measurements of apoptotic cells using AOPI double staining

In this study, MDA-MB-231 cells were treated with MEDL and 5-FU at their respective IC_50_ concentration_,_ stained with acridine orange propidium iodide (AOPI) double staining and observed under a fluorescence microscope in three independent experiments in order to identify and quantify the cell death mode after 24, 48 and 72 h of incubation periods with treatments. The AOPI assay was conducted in order to distinguish the morphological appearances of viable cells, early apoptotic cells, late apoptotic cells and necrotic cells using a DNA-binding dye AO and PI. AO intercalates into DNA which gives green fluorescence to the viable cells, while PI is only taken up by non-viable cells which intercalate into DNA and gives an orange fluorescence (Cury-Boaventura et al. 2006). The viable cells displayed a uniform green fluorescence with the appearance of a circular cell with an intact nucleus. Early apoptotic cells have green nuclei with chromatin condensation and membrane blebbing while late apoptotic cells have orange to red nucleus with condensed or fragmented chromatin. Necrotic cells display a uniform orange to red nucleus with a condensed structure (Stankovic et al. 2011; Alabsi et al. 2012).

**Figure 7.**
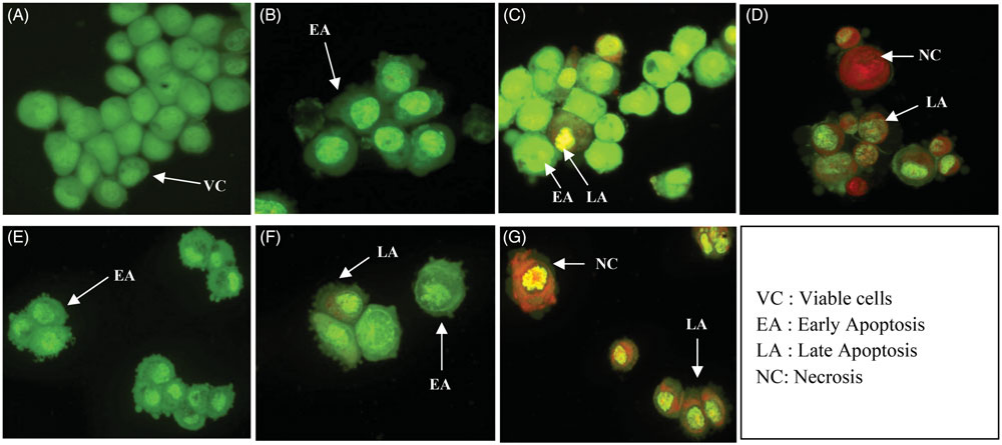
Fluorescence microscopy examination of MDA-MB-231 cells (400× magnifications). Untreated MDA-MB-231 cells (A). MDA-MB-231 cells treated with IC50 of MEDL after 24 h (B), 48 h (C) and 72 h (D). MDA-MB-231 cells treated with IC50 of 5-FU after 24 h (E), 48 h (F) and 72 h (G). Viable cells displayed the appearance of circular cells, with round and organized nuclei structure. Early apoptotic cells show visible membrane blebbing with condensed or fragmented chromatin. Late apoptotic cells have condensed or fragmented chromatin. Necrotic/secondary necrotic cells have a uniformly condensed nuclei structure. Experiment was completed in 3 independent experiments in triplicate.

As shown in Figure 7, intact nucleus and membrane are clearly seen as bright green fluorescence for the untreated MDA-MB-231 cells at 24, 48 and 72 h. However, blebbing of the cells and membrane damage were observed after 24 h of MEDL treatment indicating that the cells were in the early stage of apoptosis. At 48 h after MEDL treatment, some of the cells were stained green with the presence of membrane blebbing, and this indicated that the cells were in early apoptosis. Some of the cells had even undergone late apoptosis where the cells were stained orange with fragmented chromatin. At 72 h post treatment of MEDL, parts of the cells were in late apoptosis, and some might have undergone a necrotic/secondary necrotic state where the cells were stained with a uniformed orange and red with a condensed structure. On the other hand, MDA-MB-231 cells treated with 5-FU showed morphological characteristics similar to the cells treated with MEDL. This outcome provides qualitative morphological information that MEDL could induce apoptosis and inhibit cancer cell growth in MDA-MB-231 cell lines.

Quantitative measurements of the apoptotic cells were conducted by calculating the percentage of viable cells, apoptotic cells and necrotic cells from a total of 200 cells observed under the fluorescence microscope in three independent assays. The calculation of apoptotic cells is described as the percentage of apoptotic cells within the overall population of the cells. Table 2 shows the viable cells were reduced significantly from 75.83% to 64.17% to 46.67% at 24, 48, and 72 h, respectively. Following this, the early apoptotic cells increased significantly (*p < *0.05) from 8.17% to 8.67% to 11.83% at 24, 48, and 72 h, respectively. The late apoptotic cells were also seen to increase significantly (*p <* 0.05) from 10.67% to 11.17% to 17.50% at 24, 48, and 72 h, respectively. For the necrotic cells, the percentage was also seen to increase significantly (*p <* 0.05) from 5.50% to 16.00% to 24.00% at 24, 48, and 72 h, respectively. The results show that MEDL could induce apoptosis in MDA-MB-231 cell lines in a time-dependent manner. On the other hand, cells treated with 5-FU showed a decrement in the viable cells with values of 68.33%, 48.67% and 42.00% at 24, 48, and 72 h, respectively. The early apoptotic cells showed an increment from 24 to 48 h post incubation at 27.83% and 34.83%, respectively. However, at 72 h post incubation with 5-FU, the quantity of early apoptotic cells reduced to 12.17%. For the late apoptotic cells, it was seen that the cells increased from 2.17% to 13.17% to 17.17% at 24, 48, and 72 h, respectively. The necrotic cells showed an increment from 24, 48, and 72 h with values of 1.33%, 3.33% and 28.17%, respectively. The apoptotic features of the AOPI result were supported with the quantitative measurement of the apoptotic cells which was illustrated by the differential scoring of the treated and untreated MDA-MB-231 cells with a significant difference (*p <* 0.05) in the number of apoptotic cells in a time-dependent manner. Both morphological and AOPI assays suggest that MEDL has the ability to increase the percentage of apoptotic cells in MDA-MB-231 cells, in a time-dependent manner.

**Table 2.** Percentages of viable, apoptotic and necrotic cells after 24, 48, and 72 h treatment.

MDA-MB-231 cells and Treatment	Viable cells (%)	Early Apoptotic (%)	Late Apoptotic (%)	Necrotic (%)
Untreated cells after 24 h	93.17 ± 0.88	2.33 ± 0.17	1.67 ± 0.67	2.33 ± 0.33
MEDL treated cells after 24 h	75.83 ± 0.88^a^	8.17 ± 0.44^a^	10.67 ± 0.33^a^^b^	5.50 ± 0.50^a^^b^
5-FU treated cells after 24 h	68.33 ± 0.44	27.83 ± 0.60	2.17 ± 0.17	1.33 ± 0.33
Untreated cells after 48 h	88.83 ± 0.73	4.17 ± 0.44	3.33 ± 0.17	3.67 ± 0.33
MEDL treated cells after 48 h	64.17 ± 1.17^a^	8.67 ± 0.88^a^	11.17 ± 0.93^a^	16.00 ± 0.58^a^^b^
5-FU treated cells after 48 h	48.67 ± 1.36	34.83 ± 0.73	13.17 ± 1.33	3.33 ± 0.44
Untreated cells after 72 h	84.33 ± 0.44	6.83 ± 0.17	3.50 ± 0.50	5.33 ± 0.17
MEDL treated cells after 72 h	46.67 ± 0.60^a^	11.83 ± 0.33^a^	17.50 ± 0.87^a^	24.00 ± 0.58^a^
5-FU treated cells after 72 h	42.00 ± 0.29	12.17 ± 0.60	17.67 ± 0.44	28.17 ± 0.44

Data are presented as mean ± standard error (*n* = 3).

^a^Statistically significant (*p <* 0.05) compared to the untreated cells.

^b^Statistically significant (*p <* 0.05) compared to the 5-FU treated cells.

### The effects of *D. linearis* methanol extract on cell cycle distribution

To identify whether the growth inhibitory effect of MEDL was caused by specific disruption of the cell cycle-related event, the DNA content of MDA-MB-231 cells were measured using a flow cytometric analysis. Flow cytometry was used to monitor cell cycle distribution in MDA-MB-231 cells after exposure to MEDL at IC_50_ concentration for 24, 48, and 72 h. Measurements of the cell cycle analysis were conducted by calculating the proportion of cells in each cycle from a total of 10,000 cells in three independent assays. Flow cytometry also enables the identification of cell distribution in diverse phases of cell cycle. There are four distinct phases that could be distinguished in a proliferating cell population: the G_1_, S (DNA synthesis), G_2_ and M phase (mitosis) (Nunez 2001).

**Figure 8.**
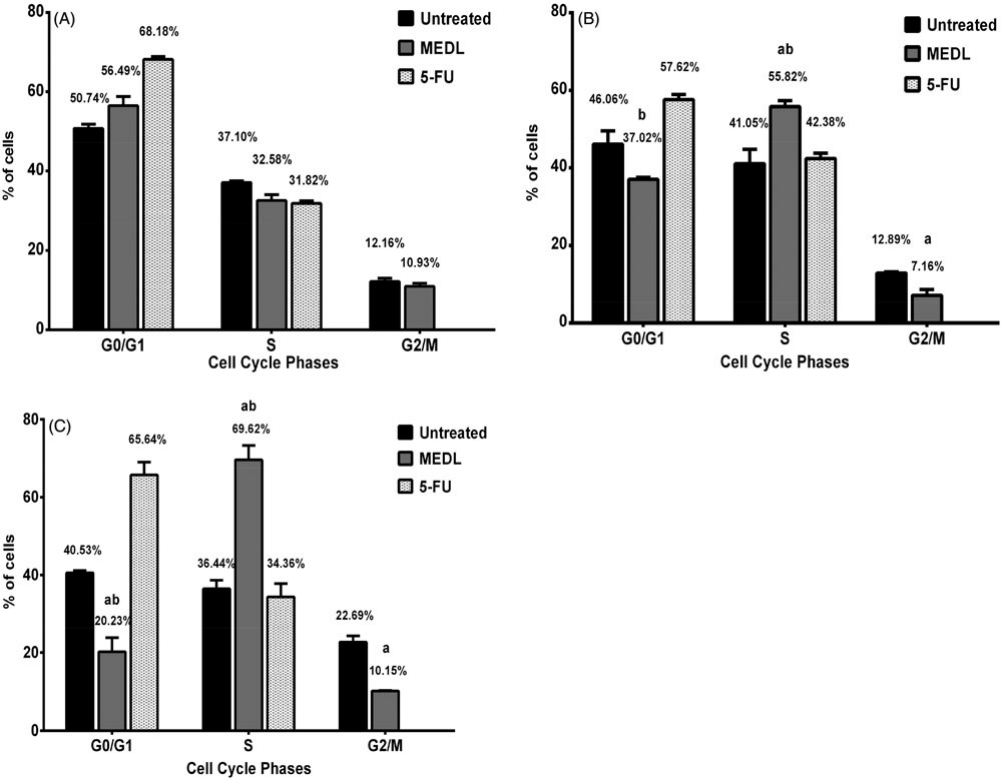
Record of flow cytometry data for cell cycle analysis. The phases of the cell cycle were determined in MDA-MB-231 cells following treatment with MEDL for 24 h (A), 48 h (B) and 72 h (C) in comparison to the untreated cells and 5-FU treated cells. Data are presented as mean ± standard error (*n* = 3). ^a^Statistically significant (*p <* 0.05) compared to the untreated cells. ^b^Statistically significant (*p <* 0.05) compared to the 5-FU treated cells.

Figure 8 shows the MEDL treated cells in G_0_/G_1_ phase which was reduced from 56.49% at 24 h to 37.02% at 48 h and later to a significant reduction at 20.23% at 72 h when compared to the untreated cells. As the percentage of cells in G_0_/G_1_ phase became reduced in MEDL treated cells, the cells in the S phase were seen to increase from 32.58% at 24 h to 55.82% at 48 h and 69.62% at 72 h. The proportion of cells in the S phase increased significantly compared to the untreated and 5-FU treated cells at 24, 48, and 72 h of treatment (*p < *0.05). The quantity of cells in MEDL treated cells in G_2_/M phase was reduced significantly from 10.93% at 24 h to 7.16% at 48 h when compared to the untreated cells. At 72 h of post treatment, the cells slightly increased to 10.15%; however, this was still considered as a significant reduction when compared to the untreated cells (*p < *0.05). The results indicate that MEDL has the ability to induce a significant time-dependent increase in the proportion of the S phase in MDA-MB-231 cells population. The cells in the G_0_/G_1_ phase in 5-FU treated cells showed a slight decrement from 68.18% at 24 h to 57.62% at 48 h, but at 72 h, the cells increased to 65.64%. The S phase in 5-FU treated cells showed the same pattern as the G_0_/G_1_ phase where it increased from 31.82% at 24 h to 42.38% at 48 h and reduced to 34.36% at 72 h. No cell was detected in the G_2_/M phase in the 5-FU treated cells. As shown in Figure 8, the MDA-MB-231 cells demonstrated an increase in the number of cells in the S phase in a time-dependent manner with a corresponding decrease in the proportion of G_2_/M and G_0_/G_1_ population. This suggests that the growth of the cancer cells was not progressing through the cell cycle; therefore, it is proposed that MEDL could effectively block cell divisions of the cancer cells. The MDA-MB-231 cells accumulated in the S phase and were unable to progress to the G_2_/M phase, suggesting that DNA synthesis in the MDA-MB-231 cells were interrupted (Xu et al. 2001).

### The effects of *D. linearis* methanol extract on early and late apoptosis

**Figure 9.**
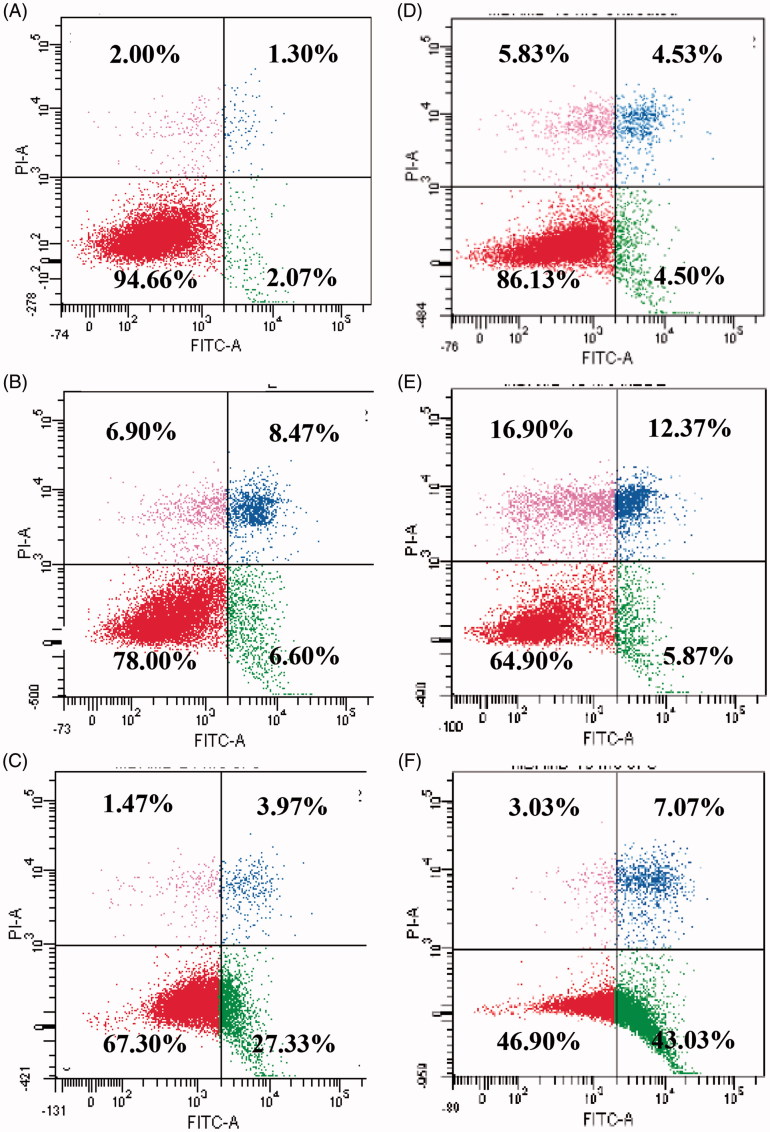
Contour diagram of annexin V/PI flow cytometry. (A) Untreated MDA-MB-231 cells after 24 h. (B) MEDL treated MDA-MB-231 cells after 24 h. (C) 5-FU-treated MDA-MB-231 cells after 24 h. (D) Untreated MDA-MB-231 cells after 48 h. (E) MEDL treated MDA-MB-231 cells after 48 h. (F) 5-FU treated MDA-MB-231 cells after 48 h.

One of the approaches to distinguish living cells in early and late apoptosis is by using a combination of fluorescein iso-thiocyanate (FITC)-labeled annexin V (Annexin V-FITC) with propidium iodide. The earliest feature of apoptosis includes loss of plasma membrane asymmetry. Initiation of apoptosis indicated by changes in cell cycle was confirmed by analysis of the externalization of phosphatidylserine (PS) using annexin V-FITC/PI staining. Phosphatidylserine, an anionic phospholipid, is normally located on the inner leaflet of healthy cells. However, during apoptosis, the PS is translocated to outer leaflet. Annexin V is a calcium-dependent phospholipid-binding protein that has high affinity for PS, therefore allowing the localization of PS in the cell being studied (Vermes et al. 1995; Engeland et al. 1996; Ziegler 2004). As a result, staining with annexin V is occasionally used in conjunction with a vital dye such as PI to discriminate the early and late apoptotic cells. Viable cells with intact membranes exclude PI; the membranes of dead and damaged cells are permeable to PI (Hingorani et al. 2011). Figure 9 shows the results of annexin V/PI flow cytometry of MDA-MB-231 cells after treatment with IC_50_ values of MEDL and 5-FU. Lower left quadrant of cytograms shows viable cells, indicating that they are annexin V-FITC and PI negative, and the lower right quadrant shows the apoptotic cells, which are annexin V-FITC positive and PI negative. The upper right quadrant shows the end stage apoptosis cells, which are annexin V-FITC and PI positive, and the upper left quadrant represents dead cells/necrotic cells, which are annexin V-FITC negative and PI positive.

**Figure 10.**
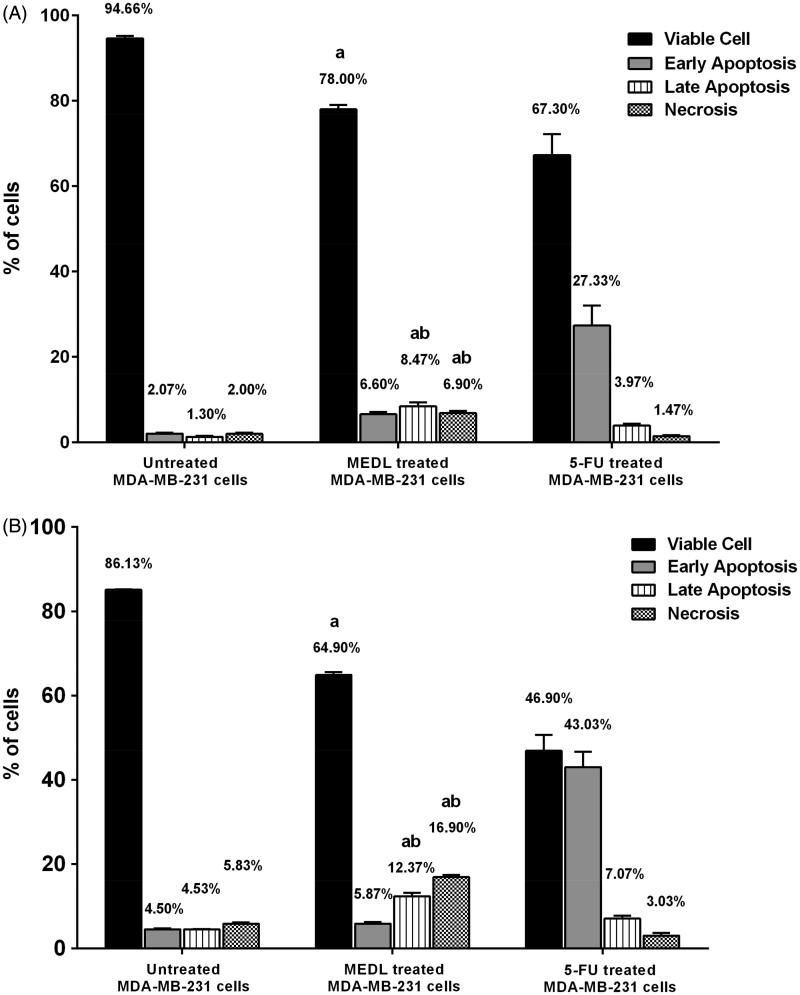
Apoptosis study of MDA-MB-231 cells exposed to MEDL at IC_50_ value. The distribution of cells undergoing early and late apoptosis together with viable and necrotic cells following treatment with MEDL for 24 h (A) and 48 h (B) in comparison with respective control using Annexin-FITC and PI flow cytometric analysis. The values are presented as mean ± standard error of mean of three determinations, and where indicated by: (A) showed a significant difference (*p <* 0.05) relative to the untreated cell; (B) showed a significant difference (*p <* 0.05) relative to the 5-FU-treated cell.

The measurements of the annexin V analysis were conducted by calculating the percentage of viable, early apoptotic cells, late apoptotic cells and necrotic cells from a total of 10,000 cells in three independent assays. Following 24 and 48 h of treatment, the proportion of viable cells treated with the IC_50_ concentration of MEDL reduced significantly (*p <* 0.05) with the values of 78.00% and 64.90% when compared to the untreated cells. However, the proportion of early apoptotic cells reduced from 6.60% to 5.87% at 24 and 48 h, respectively. Following this, the proportion of the late apoptotic cells in the MEDL-treated group at 24 and 48 h increased significantly (*p <* 0.05) with values of 8.47% and 12.37%, respectively, while the proportion of cells in necrotic/secondary necrotic cells in the MEDL-treated group was seen to increase significantly with the values of 6.90–16.90% at 24 and 48 h of treatment. Cells that were treated with 5-FU showed more compelling results. At 24 and 48 h of treatments, the proportion of viable cells decreased from 67.30% to 46.90%. Together with the decrement of the viable cells, the proportion of the early apoptotic cells reduced to 27.33% and 43.03% after 24 and 48 h of treatment (Figure 10). The proportion of late apoptotic cells showed an increment from 3.97% to 7.07% while the proportion of necrotic/secondary necrotic cells showed a slight increment from 1.47% to 3.03% at 24 and 48 h of treatment. The results indicate that the annexin V-FITC/PI staining had detected translocation of phosphatidylserine in MDA-MB-231 cells that were exposed to MEDL, signaling the induction of apoptosis. A significant increase in apoptotic cells was detected at 24 h of treatment with MEDL, and the number of apoptotic cells escalated as the incubation period with MEDL was prolonged to 48 h. Ultimately, the apoptotic cells progressed into late apoptosis and necrotic/secondary necrotic cells in the absence of phagocytes to ingest the apoptotic bodies in this study (Silva 2010).

The above data suggest that another inhibitory role played by MEDL on MDA-MB-231 cells was via the induction of apoptosis. In addition, the outcomes of annexin V-FITC/PI staining were comparable to the AOPI quantitative measurement result for apoptosis detection. The characteristics of apoptotic cells were detected by both AOPI fluorescence microscopy and annexin V-FITC/PI staining. Nevertheless, a more conclusive data should be elucidated in future studies to confirm the findings.

## General discussion and conclusions

The data from this study suggest that MEDL shows promising anti-proliferative activities against MDA-MB-231 cells via induction of apoptosis and S-phase arrest. The results can be closely related with previously reported bioactive compounds isolated from the extracts of *D. linearis*. Previous studies have identified the presence of several phytoconstituents (e.g., flavonoids, saponins, triterpenes, tannins, steroids) in the leaves of *D. linearis* (Zakaria et al. 2006, 2011; Rodzi et al. 2013; Kamisan et al. 2014). Many of these are known to possess antioxidant activity and can inhibit the growth of breast cancer cells (Ren et al. 2003; Podolak et al. 2010; Gupta et al. 2013; Katyal and Khajuria 2014; Chudzik et al. 2015; Yildirim and Kutlu 2015). Therefore, it is likely that at least some of these phytoconstituents can be ascribed to the growth inhibitory effects of the extracts used in this study.

MEDL is a flavonoid-rich extract as reported by Rodzi et al. (2013) and Kamisan et al. (2014). According to Agarwal et al. (2006), the anticancer activity of flavonoid has been associated with a modulation of cell cycle arrest at G_1_/S phase. This can lead to the apoptosis induction in breast cancer cells (Brusselmans et al. 2005). Studies conducted by Tsui et al. (2014) and Srivastava et al. (2016) proved that flavonoid induced S phase arrest during cell cycle progression. Therefore, the S phase arrest in MDA-MB-231 cell cycle progression can be related to the presence of these phytoconstituents in MEDL. At the same time, Kamisan et al. (2014) also demonstrated that MEDL possesses high amount of saponin. Based on the discovery of Russo et al. (2005) and Lemeshko et al. (2006), saponin has been proven to induce apoptosis by causing permeabilization of the mitochondrial membranes or necrotic cell death, depending on the type of cancer cells. Saponins were also reported to have cell cycle arrest induction property on MDA-MB-231 cell line (Choi et al. 2009). Other phytoconstituents from *D. linearis* were also reported to induce apoptosis in breast cancer cells include terpenoids (Bishayee et al. 2011) and tannins (Nie et al. 2016), while triterpenes from *D. linearis* was reported to have cell cycle arrest induction property on MDA-MB-231 cell line (Sathya et al. 2014).

Previous studies by Zakaria et al. (2011) and Kamisan et al. (2014) also identified that MEDL showed high DPPH and superoxide anion-radical scavenging activities as well as high total phenolic content (TPC) and oxygen radical absorbance-capacity (ORAC) values, which suggest that this extract exhibits high antioxidant capacity. The presence of these compounds in MEDL are believed to contribute in the anti-proliferative activity of MEDL through antioxidant and free radical scavenging effects, as evidenced in other studies (Zakaria et al. 2011; Baharum et al. 2014). Therefore, it is anticipated that the presence of various phytoconstituents in MEDL, coupled with its high antioxidant activity might be the reason that could be related to the induction of apoptosis and S-phase arrest as demonstrated in this study. They may work individually, or synergistically to produce the observed growth inhibitory effects on MDA-MB-231 cells.

In summary, this study has shown that MEDL possesses significant cytotoxic effects towards MDA-MB-231 cell line. It also shows a selective cytotoxic activity since the normal mouse fibroblast cells (3T3) were not harmed. MEDL has also successfully induced cell death via apoptosis in MDA-MB-231 cell line, and the results are seen in both the morphological and AOPI staining assays. MEDL induces apoptosis in MDA-MB-231 cells in a time-dependent manner. In addition, MEDL has also been proven to play a role in cell cycle progression by inducing the S phase arrest in MDA-MB-231 cells in a time-dependent manner. In conclusion, MEDL is postulated to be a promising novel source for the treatment of breast cancer, and further investigation is needed to delineate its possible anticancer property and to isolate the responsible bioactive compound from *D. linearis*.
